# Examining the Role of a Functional Deficiency of Iron in Lysosomal Storage Disorders with Translational Relevance to Alzheimer’s Disease

**DOI:** 10.3390/cells12222641

**Published:** 2023-11-16

**Authors:** Steven M. LeVine

**Affiliations:** Department of Cell Biology and Physiology, University of Kansas Medical Center, Kansas City, KS 66160, USA; slevine@kumc.edu

**Keywords:** Alzheimer’s disease, Gaucher’s disease, iron, Krabbe’s disease, lysosome, mucolipidosis type IV, neuronal ceroid lipofuscinosis, Niemann–Pick type C disease

## Abstract

The recently presented Azalea Hypothesis for Alzheimer’s disease asserts that iron becomes sequestered, leading to a functional iron deficiency that contributes to neurodegeneration. Iron sequestration can occur by iron being bound to protein aggregates, such as amyloid β and tau, iron-rich structures not undergoing recycling (e.g., due to disrupted ferritinophagy and impaired mitophagy), and diminished delivery of iron from the lysosome to the cytosol. Reduced iron availability for biochemical reactions causes cells to respond to acquire additional iron, resulting in an elevation in the total iron level within affected brain regions. As the amount of unavailable iron increases, the level of available iron decreases until eventually it is unable to meet cellular demands, which leads to a functional iron deficiency. Normally, the lysosome plays an integral role in cellular iron homeostasis by facilitating both the delivery of iron to the cytosol (e.g., after endocytosis of the iron–transferrin–transferrin receptor complex) and the cellular recycling of iron. During a lysosomal storage disorder, an enzyme deficiency causes undigested substrates to accumulate, causing a sequelae of pathogenic events that may include cellular iron dyshomeostasis. Thus, a functional deficiency of iron may be a pathogenic mechanism occurring within several lysosomal storage diseases and Alzheimer’s disease.

## 1. Introduction

**Figure 1:**
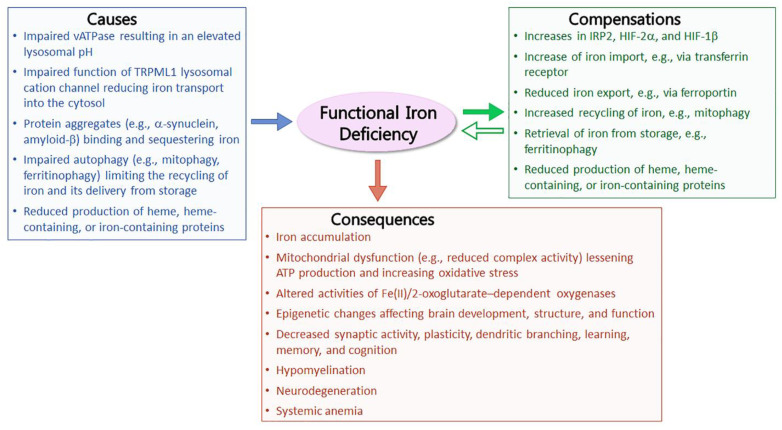
The development and implications of a functional iron deficiency within several lysosomal storage diseases and Alzheimer’s disease. Various pathogenic mechanisms can lessen the availability of iron (blue box). When available iron levels fall, cells respond by various means: attempting to acquire more iron, limiting the export of iron, increasing the release of iron from stores (e.g., ferritinophagy), and increasing the recycling of iron (e.g., mitophagy), etc. (green box). If these responses are sufficient to compensate for the decrease in available iron, then the cell can avoid negative consequences. If the compensation is insufficient, then cells would experience a functional deficiency of iron (open green, left-facing arrow), which can have a range of consequences including, but not limited to, hypomyelination, decreased plasticity, and neurodegeneration (brown box).

An increase in the level of iron within the CNS occurs across numerous neurological diseases. The underlying mechanisms for this elevation have not been established. However, an often overlooked premise is that the increase in the amount of iron could be the result of the brain needing more iron [1]. For instance, if some iron becomes unavailable for biochemical reactions, it can decrease the amount of available iron needed for cellular functions. Thus, as the amount of unavailable iron increases, it compels cells to compensate by taking up more iron, causing the total amount of iron to increase (Figure 1). Thus, it is possible to have a functional deficiency of iron in the context of elevated levels of iron.

Although iron is an essential element for cellular functions, an increased level of total iron has the potential to amplify the oxidative burden or contribute to other pathological processes, such as promoting the aggregation of proteins (e.g., tau or α-synuclein) or peptides (e.g., amyloid β) [2,3,4,5]. On the other hand, an iron-deficient state can lead to an increase in mitochondrial oxidative damage [6], and disrupt cellular activities, such as mitochondrial respiration and motility within dendrites [7,8]. Impaired mitochondria function can decrease the production of ATP [9,10,11], the main energy source for neurons whose activity disproportionately elevates the demand for ATP within the brain compared to other organs [12]. 

Alongside impaired energy production, dysfunctional mitochondria can lead to additional consequences, e.g., decreasing the production of molecules necessary for a range of cellular processes such as lipid synthesis [13], elevating the oxidative burden [14,15], and possibly increasing the sensitivity to various cell death pathways, such as glutamate excitotoxicity [16,17,18,19]. An elevated oxidative burden can cause damage to nucleic acids (e.g., mitochondrial and nuclear DNA), lipid peroxidation, etc., which have been observed in neurodegenerative diseases, such as Alzheimer’s disease and in its presumed precursor, mild cognitive impairment [20], and glutamate excitotoxicity is considered to have a contributory role in Alzheimer’s disease, particularly in moderate to advanced stages [21,22].

In addition to mitochondrial function, other iron-related processes and iron-dependent enzymatic activities may be affected when available iron is depleted, such as enzymes for myelin synthesis (or remyelination) and Fe(II)/2-oxoglutarate-dependent (Fe/2OG) oxygenases [23,24,25]. If a functional iron deficiency affects the activities of the family of Fe/2OG oxygenases, then there can be widespread implications since these enzymes are involved with transcription regulation, repair of nucleic acids, oxygen sensing, lipid metabolism, etc. [25]. Eventually, altered levels and/or activities of proteins can impact a variety of CNS functions, including decreased synaptic activity, impaired dendritic growth, diminished learning, and even neuronal death [1]. 

The concept of a functional iron deficiency was developed based on new findings and re-analyses of the scientific literature pertaining to Alzheimer’s disease [26]. In Alzheimer’s disease, the reasons why iron can become unavailable can be multifold, including iron-binding protein aggregates such as amyloid β or tau, iron not undergoing proper transport from the lysosome to the cytosol, impaired recycling of iron-rich structures (ferritinophagy and mitophagy), and altered catabolism or production of heme, iron-containing proteins, or proteins linked with iron-related processes [1] (Figure 1). This pathogenic process was named the Azalea Hypothesis of Alzheimer’s Disease, due to similarities in the causes and consequences between chlorosis in azaleas and those for a functional iron deficiency in Alzheimer’s disease [1]. Upon examination of other neurological diseases, it is apparent that many also have features that are consistent with a functional iron deficiency. One category of diseases with these features are lysosomal storage disorders. Here, the evidence supporting a functional iron deficiency was examined for various lysosomal storage diseases, i.e., mucolipidosis type IV, Niemann–Pick type C disease, neuronal ceroid lipofuscinosis, Gaucher’s disease, and Krabbe’s disease. In addition, the features of a functional iron deficiency that are shared between lysosomal storage diseases and Alzheimer’s disease are discussed.

## 2. Features of a Functional Iron Deficiency

A functional deficiency is thought to encompass many features, including abnormal iron accumulation (or iron deposits) within regions of the CNS, transcriptome and proteome changes reflecting an attempt to increase iron uptake and/or decrease iron export from cells in the CNS, disrupted lysosomal function (e.g., high lysosomal pH disrupting iron transport), impaired autophagy (e.g., mitophagy or ferritinophagy), altered proteostasis, and impaired mitochondrial function (e.g., the activity complexes) [1] (Figure 1). The greater number of affected features, and the degree of the disruption within features, likely relate to the relative impact that a functional iron deficiency has on the disease course. Many changes (e.g., transcriptome and proteome) would likely be most readily observed during the early stages of disease before extensive neurodegeneration or within select cells at later stages, which have other pathological features, such as gliosis. Some diseases may have a critical developmental window (e.g., during active myelination and/or early brain development) where a functional iron deficiency has particular relevance. For other diseases, this pathogenic mechanism may be insidious and preferentially affects post-mitotic cells, such as neurons. Depending on the severity and timing, a functional iron deficiency could decrease enzymatic activities (e.g., mitochondrial complexes), impair brain development, diminish higher brain functions, and cause neurodegeneration [1,26,27,28].

## 3. Lysosomal Storage Diseases and Iron

### 3.1. Overview of Iron Perturbations in Lysosomal Storage Diseases

Lysosomal storage diseases often have devastating consequences. The range of presentations include early onset and death in early childhood to adult onset with neurological disabilities. Although lysosomal storage diseases result in impaired digestion of substrates, there can be various affected organelles [29] and several associated pathogenic mechanisms, such as inflammation and disrupted proteostasis [30,31]. In addition, multiple cellular processes can be impaired, e.g., autophagy, calcium homeostasis, endocytosis, and synaptic function [32]. Given that the lysosome has a central role in maintaining iron homeostasis within the cell (discussed below), it raises the possibility that a lysosomal storage disease can also lead to a disturbance of cellular iron homeostasis.

**Figure 2:**
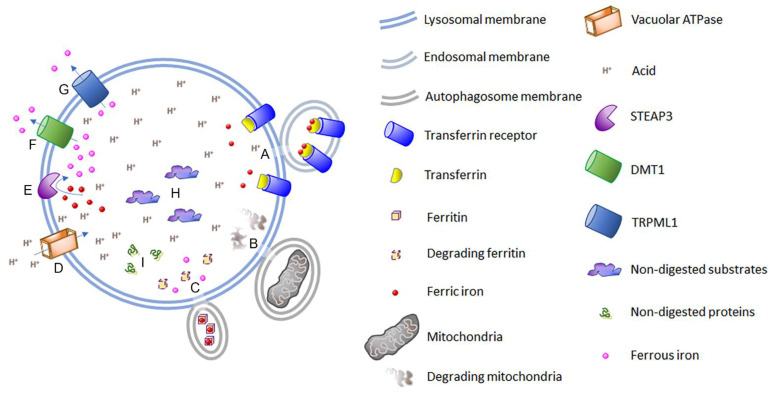
The role of the lysosome in cellular iron homeostasis and in the development of a functional iron deficiency. (**A**) The iron–transferrin–transferrin receptor complex undergoes endocytosis. Following endosome fusion with the lysosome, the complex becomes exposed to the acidic lysosomal milieu, resulting in ferric iron being released. Transferrin and its receptor then undergo recycling [43]. (**B**) Mitophagy is elevated during iron deficiency and is a mechanism to recycle iron [44,45]. Defects to mitophagy can occur in Niemann–Pick type C1 disease, Gaucher’s disease, and Alzheimer’s disease. (**C**) Ferritinophagy is increased during iron deficiency as a compensation mechanism, i.e., to increase the amount of available iron. Ferric iron is stored in ferritin, and upon release it is converted into ferrous iron [46,47], although some have identified ferric irons as being released [36]. (**D**) The v-ATPase pumps protons into the lysosome to generate an acidic environment, which is necessary for iron release from transferrin, STEAP3 reduction of ferric iron to ferrous iron, and for optimal functioning of various lysosomal enzymes [36]. Dysfunctional v-ATPase is observed in CLN1 and familial Alzheimer’s disease. An elevated lysosomal pH is also observed in mucolipidosis type II, and possibly type IV, as well as in sporadic Alzheimer’s disease. (**E**) STEAP3 reduces ferric iron to ferrous iron, which enables it to be transported out of the lysosome. An acidic pH is required for this reduction; otherwise, iron obtained from transferrin, ferritinophagy, and mitophagy may not become available within the cytosol, i.e., it is unable to exit the lysosome [36]. (**F**) DMT1 allows for the transport of ferrous iron and other divalent cations from the lysosome to the cytosol in exchange for a proton [34]. (**G**) TRPML1 is a cation channel that allows various cations, including ferrous iron and calcium, to enter the cytosol from the lysosome. This channel also has a role in autophagy and trafficking of vesicles [48]. The TRPML1 channel is dysfunctional in mucolipidosis type IV due to homozygous mutations in the *MCOLN1* gene. (**H**) Depending on the disease (e.g., type of lysosomal storage disease or Alzheimer’s disease), various substrates of lysosomal enzymes are not properly digested and can accumulate within the lysosome. This non-digested material can cause cellular dysfunction, e.g., decrease mitochondrial activity, impair cellular iron homeostasis, etc. (**I**) Proteolysis can become impaired in several lysosomal storage diseases, resulting in protein accumulation, e.g., α-synuclein aggregates in Gaucher’s and Krabbe’s diseases. Disrupted proteolysis also occurs in Alzheimer’s disease, e.g., amyloid β deposits. Both α-synuclein and amyloid β can bind and sequester iron.

There may be various mechanisms by which iron is normally transported among different cells of the CNS [33]. In a primary mechanism thought to occur in neurons, iron is transported from the iron–transferrin–transferrin receptor complex to the lysosome via endocytosis (Figure 2A). Once in the lysosome, the acidic pH, established mostly via the vacuolar ATPase (v-ATPase) (Figure 2D), and STEAP3 (Figure 2E), a ferricreductase, converts ferric iron to ferrous iron, enabling it to be delivered to the cytosol via the DMT1 transporter (Figure 2F) or TRPML1 channel (Figure 2G) [34,35,36,37]. Of note, the TRPML1 channel (*MCOLN1* gene) has been linked to Lewy body disease [38], and *MCOLN1* mutations cause the autosomal recessive lysosomal storage disorder mucolipidosis type IV [39]. Additional channels (e.g., Zip8 and Zip14) may also help with the uptake or cytosolic delivery of iron [40,41,42]. 

Alongside being instrumental for the delivery of endocytosed iron to the cytosol, the lysosome is involved with the recycling of iron via mitophagy (Figure 2B) and ferritinophagy (Figure 2C) [37]. Numerous other proteins are involved with endosomal, lysosomal, and autophagy processes. For instance, a deficiency in Sortilin-related receptor 1 (SORL1) was found to perturb these processes [49,50], indicating that it could also influence iron homeostasis. Indeed, zebrafish with deficient SORL1 exhibited an altered expression of genes related to iron homeostasis within the brain [51]. Of note, SORL1 has been associated both genetically and experimentally with Alzheimer’s disease [52], and the expression of its transcript was upregulated within the olfactory bulb of patients with early Alzheimer’s disease activity, perhaps as a compensatory mechanism [26]. 

Given that undigested substrates in lysosomal storage diseases have a propensity to disrupt various functions of lysosomes (discussed below), it plausible that both the uptake and recycling of iron are perturbed, which can limit the availability of iron for cellular use. But the relative impact this disturbance has on the course of the disease is not known and predicted to be highly variable between diseases. Lysosomal storage disorders have various subtypes, and a functional iron deficiency would likely be more readily observable and have a greater impact during key developmental windows or in a disease subtype evolving more slowly. It is also possible that a functional iron deficiency has a role in carriers of alleles which provide a predisposition to other neurological conditions, e.g., *GBA1* gene variants for Parkinson’s disease. Even if the role for a functional iron deficiency within a particular lysosomal storage disease is outweighed by other pathogenic processes, reviewing the factors that align with this mechanism is useful as it can provide insights about the evolution and role of disrupted iron homeostatic processes in other neurological conditions, such as Alzheimer’s disease. 

### 3.2. Mucolipidosis Type IV—Highlight of Several Pathological Features

Mucolipidosis type IV is caused by autosomal recessive mutations in the *MCOLN1* gene. The initial presentation usually occurs before one year of age and can cause both CNS and systemic pathology [39,53]. As individuals age, there are developmental delays and progressive deficits in motor function and vision [53]. In the CNS, hypomyelination and brain iron deposits are revealed via MRI [53]. Other pathologies, such a retinal dystrophy and degeneration of the cerebellum, often develop over time [53]. 

Iron deposition in the basal ganglia was present in a patient at 35 weeks of gestation [54] and found in the basal ganglia and thalamus of patients ranging in age from 16 months to 22 years [53,55]. In addition, iron deficiency with or without anemia was also observed in many patients, which was thought to be due to an impaired dietary absorption of iron [56,57]. 

### 3.3. Mucolipidosis Type IV Causes the Dysfunction of TRPML1, a Lysosomal Channel for Cations Including Fe^2+^

The *MCOLN1* gene encodes the transient receptor potential mucolipin 1 (TRPML1) channel (Figure 2G), which spans the lysosomal membrane and whose function is augmented at a low pH [58,59]. The TRPML1 channel allows for the permeability of numerous cations, including iron and calcium. Defective TRPML1 can result in a diminished efflux of iron and calcium from the lysosome, resulting in an elevated level of ferrous iron within the lysosome, as well as a reduced level of ferrous iron within the cytosol of patient fibroblasts [39,60]. The TRPML1 channel’s activity, i.e., channel currents, can be inhibited via a low pH, but this regulation is lost by some diseases, causing *MCOLN1* mutations [61]; however, in other studies, a low pH did not affect the transit of iron through the TRPML1 channel; in fact, it was increased [59,60]. There has been a debate as to whether the lysosomal pH is increased or overly acidified in mucolipidosis type IV cells [62,63,64]. An increased lysosomal pH can be present in some other lysosomal storage diseases [65,66], e.g., in skin fibroblasts from patients with mucolipidosis type II [67], which results from a deficiency in N-acetylglucosamine-(GlcNAc) 1-phosphotransferase activity. Alongside potentially favoring iron retention within the lysosome, thereby contributing to a functional iron deficiency, an elevated lysosomal pH can impair the activity of various lysosomal enzymes and possibly perturb calcium homeostasis, e.g., through TRPML1 disruption [66,68]. 

In mouse embryonic *MCOLN1*^−/−^ fibroblasts, the expression of the transferrin receptor was elevated, while ferritin light chain was reduced compared to matching wild-type cells [36]. These results indicate, respectively, that the cells were trying to take up iron and that there was not a sufficient cytosolic level of iron to put into storage. Furthermore, the deficient cells had impaired mitochondrial respiration and reduced levels of transcripts and proteins involved with mitochondrial function, which is also in alignment with a functional iron deficiency [36]. 

In retinal pigment epithelial cells with TRPML1 knockdown via siRNA, mitochondrial changes were apparent (i.e., reduced length, network, and membrane potential), which were worsened by excess iron (except for the mitochondrial network) and thought to be due to the iron-catalyzed formation of reactive oxygen species [69]. An alternative explanation is that the trapped iron within lysosomes caused the mitochondria to become deficient in iron, and these dysfunctional mitochondria resulted in elevated oxidative damage [1,6]. Supporting this notion, the addition of iron lowered lipid peroxidation in TRPML1-deficient cells, but this difference did not reach significance [69]. 

### 3.4. Mucolipidosis Type IV Impairs Myelination

As mentioned previously, hypomyelination can be detected in patients with mucolipidosis type IV disease via MRI [53]. In homozygous *MCOLN1* knockout mice, there was a reduced level of myelination [70]. One suggestion provided for this finding was due to an impaired delivery of iron from the lysosome to the cytosol, resulting in functionally deficient levels of iron needed for myelination [70]. In a subsequent study, it was implied that iron mishandling was oligodendrocyte specific, occurred during a developmental window of myelination, or that iron was partially sequestered within lysosomes [71]. Of note, the excess iron contained within lysosomes may also promote the accumulation of lipofuscin in post-mitotic cells, such as neurons [58,60]. 

### 3.5. The Potential Impact of a Functional Iron Deficiency in Mucolipidosis Type IV

Several pieces of evidence support a role for a functional deficiency of iron in mucolipidosis type IV, but it may be targeted via a cell type and/or development window. For instance, iron is usually present in copious amounts in oligodendrocytes [72,73] and is required for myelination [23,74], which begins during gestation and is robust during the first two decades of life [75,76]. If iron is inaccessible, or deficient, for biochemical reactions during early life, then this could lead to hypomyelination [23]. 

Elevated levels of iron deposition in subcortical gray matter structures can be an indication that iron is unavailable in these regions, causing the brain to respond to acquire more iron [1]. More slowly developing gray matter pathology that occurs in this disease could indicate that a restricted availability of iron has a pernicious pathogenic role in neurons, which are post-mitotic and therefore unable to renew themselves. Of note, *MCOLN1* gene variants have been linked to Lewy body disease [38]. If this connection to Lewy body disease is confirmed, then it helps establish a pathogenic role for this lysosomal channel (and therefore lysosomal dysfunction) in other neurodegenerative diseases.

### 3.6. Niemann–Pick Type C Disease, Gangliosides, and GM2 Gangliosidosis

Niemann–Pick type C disease causes the deposition of glycosphingolipids and cholesterol within endosomes and lysosomes [77,78], and consistent with this, there is an autophagic impairment in *NPC1*^−/−^ neurons [79], including defective mitophagy in neuronally differentiated fibroblasts with *NPC1* or *NCP2* mutations [80]. Impaired mitophagy can interfere with the recycling of iron (Figure 2B), which could lower the amount of available iron. Interestingly, GM2 ganglioside, GM3 ganglioside, and other glycosphingolipids accumulate within the neurons of *NPC1*^−/−^ mice, and the accumulation of cholesterol in neurons is reliant on gangliosides, particularly GM2 [81,82]. In a mouse model of GM2 gangliosidosis, i.e., with disruption of the *HEXB* gene (a model of Sandhoff disease), there was an increase in the transferrin receptor in the brain and spinal cord [83]. Since this receptor is used for iron uptake (Figure 2A), this indicates that cells in the CNS of these mice required more iron and were compensating accordingly. Neurons from the *HEXB*^−/−^ mice displayed various alterations (swollen lysosomes, dilated mitochondria, fewer mitochondria, etc.) and administration of iron slowed the disease course [83], which support the prospect that there was a functional iron deficiency.

### 3.7. Disturbances to Iron Homeostasis in Niemann–Pick Type C Disease

In Niemann–Pick type C1 (*NPC1* gene) disease, 7T MR brain T1 imaging and quantitative susceptibility mapping revealed iron accumulation in the pulvinar nucleus, which was correlated with disease activity [84]. Biochemical measures revealed a possible trend for iron accumulation in the cerebellum and decreased levels of iron within the CSF and plasma of patients with type C1 disease [85]. Furthermore, iron levels were elevated in the cerebellum and cerebrum, and decreased in the liver, in a mouse model of Niemann–Pick type C1 disease (*NPC1*^−/−^*)* [85]. Interestingly, treatment of these mice with the iron chelator deferiprone did not improve the lifespan, and a high dose of the chelator even worsened several disease parameters, indicating that a deficiency of iron contributed to the decline [86]. On the other hand, iron chelation lessened iron accumulation, reversed an autophagic defect, and enhanced the lifespan in a yeast model of Niemann–Pick type C1 disease, indicating that the accumulated iron caused toxicity in this model [87]. Interestingly, the same level of iron chelation reduced the lifespan of wild-type yeast to a level similar to that attained in the defective yeast given iron chelation, indicating that limiting iron impaired mitochondrial activity in normal yeast [87].

Ferritin levels across a range of tissues were found to be decreased in Niemann–Pick type C1 disease, and it was suggested that this was due to iron being retained within lysosomes and therefore not available for storage into ferritin [88]. In NPC1-deficient auditory cells, iron levels increased but the levels of both the transcript and protein for the transferrin cellular receptor increased [89], indicating that the cells were trying to acquire additional iron consistent with a functional iron deficiency. Also consistent with less available iron, an elevated level of iron regulatory protein 2 mRNA and protein were observed in NPC1-deficient cells [89]; note, an elevated level of iron normally results in the degradation of iron regulatory protein 2 [90]. Furthermore, a lower level of ferritin was also observed [89], indicating less available iron to put into storage. Alternatively, it was put forward that less ferritin was accounted for by an increase in ferritinophagy associated with elevated expression of NCOA4 [89], which is a cargo receptor used to target ferritin to the lysosome [91]. However, an increase in ferritinophagy is a mechanism used to help supply the cell with more available iron [92] (Figure 2C), which supports the notion that these cells were experiencing a functional deficiency of iron. Furthermore, mitochondrial changes were noted in NPC1-deficient auditory cells [89], which is also consistent with a functional iron deficiency. 

Alterations were observed across a variety of iron-related parameters in samples from both mice and humans with Niemann–Pick type C1 disease [93]. Examples of parameters that were affected include lower hematocrit, lower corpuscular hemoglobin and volume, lower levels of iron and light chain ferritin, and increased levels of soluble transferrin receptor in the serum of affected mice [93]. Many of these parameters had a similar direction of change between affected mice and human patients, with some in the latter group being at the low side of the normal range [93]. These findings indicated that Niemann–Pick type C1 disease causes dysregulation of iron homeostasis, including hematological alterations [93]. Consistent with these findings, a 2-year-old patient with Niemann–Pick type C had low blood iron with microcytosis [94]. Although microcytosis was thought to be a result of anemia of chronic disease [94], it is possible that it was more directly tied with the pathophysiology, e.g., similar to that discussed previously, leading to a deficiency in iron. Furthermore, the patient had a delay in myelination [94], which can be a feature of this disease [95] and is observed in other lysosomal storage disorders.

### 3.8. Alterations of Iron Homeostasis in Neuronal Ceroid Lipofuscinosis

Autosomal recessive mutations in the gene *PPT1*, which encodes for palmitoyl protein thioesterase 1, cause neuronal ceroid lipofuscinosis-1 (CLN1), which is one of fourteen types. In mouse models of this form of disease, as well as in other versions of disease (i.e., CLN5 and CLN6), there was an accumulation of metals, including iron, in some brain regions [96,97]. However, the level of iron declined in the retinal inner segments in CLN6 mice [98]. In CLN1 mice, there was deficient palmitoylation of the v-ATPase V0a1 subunit, causing its impaired trafficking to the lysosome. This resulted in decreased lysosomal v-ATPase activity in the brain and an elevation of the lysosomal pH in neurons [99] (Figure 2D). 

Importantly, an elevated lysosomal pH (impaired acidification) has been linked with the generation of a deficiency of iron within cells (observed within mitochondria and the cytosol) [36]. Inhibition of v-ATPase leads to iron becoming trapped in the lysosome, which is thought to be due, in part, to the inability of STEAP3 to reduce ferric iron to ferrous iron, which is needed for its transport via DMT1 or TRPML1 into the cytosol [36] (Figure 2D–G). In fibroblasts treated with an inhibitor of v-ATPase, both mitochondrial biogenesis and function become impaired, and the expression of the transferrin receptor is elevated [36]. This treatment can lead to cell death via a non-apoptotic mechanism [36].

Adult-onset neuronal ceroid lipofuscinosis is usually due to autosomal dominant mutations in the *DNAJC5* gene (CLN4), which encodes a co-chaperone cysteine string protein-α [100]. Disease-producing defects in this protein can prevent its palmitoylation. This can cause a misloading of iron–sulfur clusters onto iron–sulfur cluster assembly scaffolding proteins, resulting in oligomerization of the co-chaperone that leads to impaired synaptic mechanisms [101]. However, these results also raise the possibility that iron–sulfur clusters do not undergo proper assembly or delivery to target proteins. In iron-deficient cells, mitochondria displayed impaired gluconeogenesis, lower cytochrome concentrations, changes to their morphology, and damage [102]. Furthermore, impaired formation of iron–sulfur complexes in the mitochondria can conceivably induce a functional deficiency of iron within the cytoplasm, resulting in efforts to increase the cellular uptake of iron [103]. 

### 3.9. Gaucher’s Disease—A Deficiency of Glucosylceramidase

Autosomal recessive mutations in the gene for glucosylceramidase beta (*GBA1*), which is also known as glucocerebrosidase, decrease the enzymatic function, resulting in Gaucher’s disease. There are various subtypes of this disease (three major and two other), with two of the major subtypes (types 2 and 3) primarily affecting the nervous system, with type 2 progressing rapidly (i.e., death from 2–4 years) and type 3 progressing more slowly [104]. Although type 1 causes pathology in the periphery (such as hepatosplenomegaly, anemia, bone diseases, etc.), it does not principally affect the CNS [104]. However, patients with type 1 disease do have Gaucher cells within their CNS and a predisposition for other neuropathological changes (discussed below). 

Glucosylceramidase is a lysosomal enzyme, and deficient activity limits the degradation of the glucosylceramide and glucosylsphingosine to ceramide and glucose, and to sphingosine and glucose, respectively [104,105]. The accumulation of glucosylsphingosine is believed to be a neurotoxic metabolite that interferes with the formation and function of lysosomes, as well as triggering other pathological changes, such as elevating the mobilization of calcium (observed from brain microsomes), which may be relevant for mechanisms leading to cell death [106,107,108,109,110].

#### 3.9.1. Gaucher’s Disease, α-Synuclein, Mitochondria, and Iron

Allelic variants of *GBA1* increase the risk for Parkinson’s disease and Lewy body dementia in heterozygous carriers, and some patients with type 1 Gaucher’s disease can develop clinical and pathological features of these diseases [105,111]. Consistent with these observations, deficient glucosylceramidase can lead to the formation of α-synuclein aggregates, which are a main component of Lewy bodies that are present in Gaucher patients that develop Parkinson’s disease or Lewy body dementia [105,111,112,113]. In addition, α-synuclein oligomers and deposits were observed in the midbrain and hindbrain, respectively, of mice deficient in glucosylceramidase [114]. 

The undigested glucosylsphingosine and glucosylceramide, and other lipid metabolites (Figure 2H), can facilitate the accumulation and aggregation of α-synuclein [110,115]. Decreased activities of the lysosomal enzymes β-galactosidase, hexosaminidase, and sulfatase were observed in Gaucher’s and Parkinson’s disease patient cell lines of induced pluripotent stem cell-derived midbrain neurons, and these reductions were reversed via knockdown of α-synuclein, providing support of a pathogenic role for α-synuclein [116]. Both α-synuclein and/or misfolded glucosylceramidase can induce the unfolded protein response and cause other perturbations to the cell, such as disrupting lysosomal function, including defective autophagy and mitophagy (Figure 2B,I), and impairing the activity and delivery of hydrolases from the endoplasmic reticulum to the lysosome [105,110,111,114,117]. Note, disruption to mitophagy, induced by α-synuclein and/or misfolded glucosylceramidase, could diminish the amount of recycled iron available to the cell [1] (Figure 1).

Interestingly, iron binds α-synuclein, which affects its conformation and may promote its aggregation [118,119,120,121]. Iron also accumulates within Lewy bodies, i.e., in Parkinson’s disease [122,123]. It has been proposed that α-synuclein affects transferrin–transferrin receptor iron uptake by acting as a ferrireductase [124]. Furthermore, upon iron binding to an iron regulatory protein on the iron-responsive element in the 5′-UTR of the α-synuclein transcript, it allows for increased production of the protein [125]. Thus, there may be an interconnection between α-synuclein and elevated iron levels, with some of the iron being unavailable (i.e., bound to α-synuclein or sequestered within Lewy bodies), which would potentially limit or lower the amount of available iron.

Aggregates of α-synuclein within mitochondria may impair the function of this organelle [111]. There were reductions in the activities of the mitochondrial complexes I–III in patient-cultured fibroblasts (complex IV was not examined) [126], and a decrease in complex IV (cytochrome C oxidase) was observed in a liver of a patient, likely with type 3 disease [127]. Multiple indicators of mitochondrial health and function in the brain were decreased in models of Gaucher’s disease [114,128]. The activities of complexes I and II–III, but not IV, were reduced in the brains of mice deficient in glucosylceramidase [114], while the activities of complexes III and IV were reduced in the brains of a zebrafish model of Gaucher’s disease [129]. Glucosylsphingosine, galactosylsphingosine, and sphingosine inhibited complex IV activity in mitochondria, but not in the purified complex [130]. 

Alongside α-synuclein aggregates (or sphingosine and related molecules) inhibiting mitochondria, a decreased level of mitochondrial complex activities, particularly I and II, were observed in the spinal cord tissue of iron regulatory protein 2-deficient mice, which produce a functional iron deficiency [27]. Similarly, in systemically induced iron-deficient states, a decrease in the activities of mitochondrial complexes were observed in the heart and skeletal muscle, as well as within the hippocampus [131,132,133,134]. Thus, it is plausible that since iron can bind α-synuclein, and impaired mitophagy can lessen recycled iron, a functional iron deficiency contributes to mitochondrial dysfunction in Gaucher’s disease. 

#### 3.9.2. Gaucher’s Disease, Anemia, and Brain Development

A majority of patients with type 1 Gaucher’s disease have hyperferritinemia [135,136,137,138], and the elevated serum ferritin level could be reduced with enzyme replacement therapy [137]. Although some have suspected that hyperferritinemia may be negatively correlated with hemoglobin levels, and more pronounced in patients with anemia [135], this was not observed by others [137]. The elevated level of serum ferritin was tied to the sequestration of iron by Gaucher cells and altered metabolism of iron [137]. One mechanism put forward for the sequestration of iron by Gaucher cells was the lowered expression of the iron export protein ferroportin in these cells, which may have been due to internalization mediated by hepcidin [137]. Alongside being a pathogenic feature in the spleen, Gaucher cells are also present in the CNS, typically with a perivascular localization, in patients across all three major types of disease, although there may be fewer of these cells in type 1 disease compared to the other major subtypes [139]. 

The pediatric population of type I patients may have anemia [137]. During early life, the brain is rapidly growing, and iron is essential for this development [140]. Iron deficiency anemia is associated with impaired brain development including, but not limited to, diminished myelination, and altered structure and function of the hippocampus [23,140,141,142,143,144]. The consequences of a developmental iron deficiency can result in prolonged effects on behavior and cognition [140,141,142,143,144]. 

Iron deficiency in young rats led to lowered brain iron levels [145,146]. However, in female rats that were given an iron-deficient diet (anemia-producing diet) for two weeks prior to pregnancy, and continuing through to different endpoints, their offspring at postnatal day 10 did not have a significant decrease in the levels of iron in two regions of the brain but did have an upregulation in the transcript for the transferrin receptor [147], suggesting a compensation by the brain to preferentially take up iron. This upregulation of the transferrin receptor transcript within the brain, e.g., the hippocampus and cerebellum, during developmental iron depletion has been observed by others [148]. 

Iron depletion during development can affect the activity of iron-containing enzymes, e.g., the activity of ten-eleven translocation enzymes and possibly Jumonji C domain-containing demethylases [148,149]. This may result in altered epigenetic changes, which can impact the development of the brain with lasting consequences [148,150]. Of note, a trend for epigenetic changes has been observed in the peripheral blood of patients with type 1 Gaucher’s disease [151]. Epigenetic changes may be expected in the CNS of patients with type 1 and other forms of disease, but this is currently unknown. However, many type 1 patients have deficits in various domains of cognitive function [152,153]. This raises the possibility that the unavailability of iron may have contributed to the development of these alterations, perhaps operating through epigenetic modifications, leading to adverse effects, such as a lowering of brain-derived neurotrophic factor levels [151,154] or altering reelin levels [150], which are important for brain development and plasticity, and are linked with Alzheimer’s disease [155,156,157,158]. 

#### 3.9.3. Gaucher’s Disease and a Functional Deficiency of Iron 

There are multiple pieces of evidence indicating the involvement of a deficiency of iron promoting pathology in Gaucher’s disease (discussed above). In the periphery, anemia and/or hyperferritinemia could exert adverse consequences, particularly in very young individuals, including affecting the development and function of the brain. Within the CNS, disruption to iron homeostasis can occur several ways: Gaucher cells acquire iron, α-synuclein interacts with iron, and lysosomal function becomes altered, which in theory, could impede the delivery of iron to the cytosol. Furthermore, disrupted autophagic processes could reduce the recycling of iron. In line with these alterations, mitochondrial function is impaired (e.g., complex activities), which can be a consequence of a functional iron deficiency [1,26,27,36]. 

### 3.10. Similarities between Krabbe’s Disease and Gaucher’s Disease

Alongside having a range of disease onsets from infants to adults, Krabbe’s disease exhibits several biochemical parallels with Gaucher’s disease. In Krabbe’s disease, instead of glucosylceramidase, the lysosomal enzyme that is affected is galactosylceramidase (*GALC* gene). Thus, instead of glucosylceramide and glucosylsphingosine, the undigested substrates are galactosylceramide and galactosylsphingosine (psychosine), with the latter thought to be toxic in Krabbe’s disease [159,160]. In Krabbe’s disease, and in the spontaneously occurring mouse model called twitcher mice, the prominent pathological feature is demyelination, but numerous other pathological features are also present [161], some of which are similar to those occurring in Gaucher’s disease. For instance, instead of Gaucher cells there are globoid cells (lipid-filled, PAS-positive, multinucleated macrophages), and like Gaucher’s disease, α-synuclein aggregates occur in the brain [161,162,163]. Furthermore, a mutant *GALC* allele potentially increases the risk for developing Parkinson’s disease [164]. Thus, like Gaucher’s disease, it has been anticipated that iron would be sequestered by α-synuclein aggregates as well as in globoid cells. 

### 3.11. Krabbe’s Disease Models and Iron

In twitcher mice, the brain vasculature was observed to be enriched with iron, which was not observed in normal mice or other mouse models of dysmyelination, i.e., shiverer (myelin basic protein gene mutation), jimpy (proteolipid protein gene mutation), and quaking (chromosome 17 deletion affecting the genes *QKI*, *CAHM*, *PRKN*, and *PACRG*) [165] mice [166]. This elevated level of brain vasculature iron indicates that the brain was taking up excess iron to meet a greater demand. Of note, iron is critical for myelination, and this increase [166] was observed during a period of active myelination [23,167].

Furthermore, there was an apparent increase in the number and size of iron-rich structures within the oligodendrocyte soma, which was suggested to be iron accumulated within dysfunctional lysosomes [166], which may have similarities to that described for mucolipidosis type IV (discussed above). Deficits in proteostasis, autophagy, and mitochondrial function were also observed in models of Krabbe’s disease [168,169]. Dysfunctional lysosomes can, in theory, disrupt the transport of iron to the cytosol, while impaired autophagy can reduce the recycling of iron. The resulting lower levels of available iron can impair mitochondrial function [1,26], but psychosine, which accumulates in this disease, can also decrease cytochrome C oxidase (complex IV) activity [170].

### 3.12. The Likelihood of a Functional Iron Deficiency in Gaucher’s or Krabbe’s Disease 

As discussed previously, numerous findings have raised the prospect that a functional iron deficiency may have a contributory role to impaired systemic or CNS function in Gaucher’s disease. There is less evidence for this in Krabbe’s disease, but there are several intriguing pieces of data (e.g., iron-enriched structures in oligodendrocytes and altered autophagy) potentially supporting this disease mechanism [166,168]. It is probable that the demand for iron is increased due to disruptions in iron homeostasis in these diseases, particularly during myelination and early brain development. However, it is also possible that compensation mechanisms act to counter less available iron, and these are adequate to prevent or limit the effects of limited available iron (Figure 1). If a functional iron deficiency exerts a pathogenic role in these diseases, then chances are that it would likely be more relevant during critical developmental windows, i.e., active myelination [141]. In addition, this mechanism could participate in slower progressing forms of disease by compounding the effects of other ongoing pathogenic mechanisms. Additional evidence is needed to establish a pathogenic role for a functional deficiency of iron in one or both of these diseases or their various subtypes.

## 4. Iron in Alzheimer’s Disease and Lysosomal Storage Diseases

### 4.1. Baseline Characteristics of Lysosomal Storage Disorders and Alzheimer’s Disease Relative to a Functional Iron Deficiency 

When comparing Alzheimer’s disease and lysosomal storage disorders, it is relevant to start by recognizing that the former is a disease of the elderly (usually commencing at >65 years of age), while the latter is mostly a disease of the very young (e.g., presenting in infants or toddlers). Of course, there are exceptions; earlier-onset familial Alzheimer’s disease can start in the 4th or 5th decade of life, while several subtypes of lysosomal storage diseases have a late onset, e.g., adult-onset neuronal ceroid lipofuscinosis (CLN4) starting around the 3rd decade of life [100]. To complicate matters, there can be variable courses of disease (e.g., among the lysosomal storage diseases or their subtypes), with some rapidly progressing to death (e.g., infantile Krabbe’s disease; type 2 Gaucher’s disease), while others advance more slowly (e.g., mucolipidosis IV; type 1 Gaucher’s disease) [53,104]. Furthermore, patients that go on to develop Alzheimer’s disease would have a greater starting reserve of neuronal function than for a patient with an early form of a lysosomal storage disease when the brain is actively developing. This greater reserve may serve to slow the course, or lessen the impact, of a functional iron deficiency in patients with Alzheimer’s disease. Thus, depending on the type of lysosomal storage disease (or subtype), or the nature of the course of Alzheimer’s disease within individuals, the features and impact of a functional iron deficiency would be expected to vary. 

Important considerations are the mechanisms of disruption relative to iron homeostasis and the severity of the disruption. If there is a minimal number of pathways being affected, or the degree of the disturbance is minor, then there may be the ability to compensate for a functional iron deficiency within a lysosomal storage disease, thereby minimizing or eliminating the impact of this pathogenic mechanism (Figure 1). The ability to compensate would be expected to differ among different types of lysosomal storage disease, or their subtypes, and it would also be expected to vary within the population of patients with Alzheimer’s disease. For example, differences in *SORL1* gene alleles, and its encoded protein, which has a role in iron homeostasis, have been linked with Alzheimer’s disease [52]. 

### 4.2. Comparing the Pathways Contributing to a Functional Iron Deficiency in Lysosomal Storage Diseases and Alzheimer’s Disease

There are numerous mechanisms that can contribute to the development of a functional iron deficiency, and one or more of these may operate within several lysosomal storage diseases (Figure 1 and Figure 2). These pathways could overlap with those thought to occur during Alzheimer’s disease. A straightforward pathway that may be shared between these disease types is lysosomal acidification, or lack thereof. 

In neuronal ceroid lipofuscinosis-1, the palmitoylation of the v-ATPase V0a1 subunit is reduced, which limits its transport to the lysosome, causing decreased activity of the lysosomal v-ATPase and an elevated lysosomal pH [99]. An elevated lysosomal pH has also been observed in other lysosomal storage diseases, e.g., mucolipidosis type II [65,66,67]. Multiple links between impaired lysosomal acidification and Alzheimer’s disease pathophysiology have been previously noted [171]. In sporadic Alzheimer’s disease, the increased risk ε4 allele of *APOE* is thought to elevate the pH of lysosomes within astrocytes [172], and in Down’s syndrome the proteolytically cleaved carboxy fragment of the amyloid β precursor protein causes an elevated lysosomal pH [173,174]. In familial Alzheimer’s disease, mutations in presenilin 1 can cause the reduction in the glycosylation of the v-ATPase V0a1 subunit, thereby preventing its delivery to the lysosome [175,176,177]. With impaired lysosomal acidification, the delivery of iron to the cytosol can decline and cells can become deficient in iron [1,36]. In addition to impaired lysosomal acidification, iron is thought to become trapped within the lysosome, resulting in a functional iron deficiency due to an impairment of the TRPML1 channel (*MCOLN1* gene) [36], which occurs in mucolipidosis type IV [39].

Pathological links have been observed between lysosomal storage diseases and Alzheimer’s disease for altered processing of the amyloid precursor protein as well as lysosomal substrates (e.g., gangliosides) due to dysfunction of lysosomal and autophagic processes [178]. Disruptions to autophagy have been observed in models of lysosomal storage diseases, e.g., Niemann–Pick type C, Gaucher’s disease, and Krabbe’s disease [79,114,168]. Based on various models, autophagic defects (e.g., in mitophagy and ferritinophagy) have been inferred to occur in Alzheimer’s disease [1,36,178,179,180,181]. Thus, the recycling of iron for reuse can be inhibited via disruption to autophagy in several lysosomal storage diseases as well as in Alzheimer’s disease. 

Disruptions to the metabolism of heme are also thought to contribute to a functional iron deficiency [1]. In a mouse model of Niemann–Pick type C1 disease, there was evidence for a decreased expression of proteins involved with the synthesis, and possibly degradation, of heme in the spleen or liver [182]. Alterations to heme metabolism have been described in the context of Alzheimer’s disease [1,183,184]. 

Another mechanism thought to contribute to the development of a functional deficiency of iron is the sequestration of iron by protein aggregates. In Alzheimer’s disease, both amyloid β and tau accumulate and bind iron [1,26,185,186]. In lysosomal disorders, such as Gaucher’s disease and Krabbe’s disease, iron can bind α-synuclein, and possibly become trapped within aggregates (discussed above). The extent of this process within Gaucher’s disease and Krabbe’s disease, relative to the apparent abundant sequestration of iron in Alzheimer’s disease by amyloid β and tau, is unknown. However, within individual cells, e.g., neurons or developing oligodendrocytes [187,188], it is possible that the sequestration of iron by α-synuclein has a significant impact on cellular iron homeostasis, leading to a functional iron deficiency.

A number of lysosomal storage diseases exhibit signs in the periphery of altered iron homeostasis. For instance, in type I Gaucher’s disease, some pediatric patients may develop anemia [137], and patients with Niemann–Pick type C1 disease can display hematological alterations, implicating a dysregulation of iron homeostasis [93]. Meanwhile, a large prospective study related to Alzheimer’s disease found that anemia elevated the risk for dementia by 56% [189]. Although the brain may preferentially attempt to acquire iron at the expense of other organ systems, particularly during development [190], too little iron can impair normal brain development [23,140,141,142,143,144]. Furthermore, a functional iron-deficient state developing within the CNS, e.g., due to lysosomal dysfunction or protein aggregates, may still cause impairments, such as mitochondria dysfunction and associated neurodegeneration [1,26,27,36].

### 4.3. Shared Consequences of a Functional Iron Deficiency in Lysosomal Storage Diseases and Alzheimer’s Disease

An elevated accumulation of total iron within the CNS regions can indicate that there is not enough available iron to meet cellular needs, and the brain responds by taking up more iron [1] (Figure 1). Accumulated iron has been detected in a lysosomal storage disease. For instance, in mucolipidosis type IV, excess iron accumulation was observed in several subcortical regions, and this accumulation could begin very early, e.g., during gestation [53,54,55]. An absence of an overall accumulation of iron in a lysosomal storage disease does not necessarily indicate an absence of a functional iron deficiency. For instance, there may not have been sufficient time for the accumulation to reach levels of differential detection, or the disease progressed too rapidly, thereby exceeding the capacity by which the brain could take up extra iron. In Alzheimer’s disease, iron accumulation has been observed from early to late stages of this disease, as well as observed in both cortical and subcortical CNS regions [191,192,193,194,195]. 

There are multiple targets that have been predicted to be sensitive to a functional iron deficiency (Figure 1). For instance, mitochondrial dysfunction, e.g., decreased complex activity in the skeletal muscle and heart, can be a consequence of a systemic deficiency of iron, which can also cause reduced complex activity in the hippocampus when the deficiency arises early [28,131,132,133,134,190,196,197]. When a functional iron deficiency was generated by a disruption to iron regulatory protein 2, the activities of mitochondrial complexes I and II were subsequently diminished in mouse spinal cord tissue [27]. Impaired mitochondrial structure and function have been observed in a number of lysosomal storage diseases (Krabbe’s, Gaucher’s, GM2 gangliosidosis, and mucolipidosis type IV) [69,83,114,126,128] as well as in Alzheimer’s disease [198,199]. However, mitochondrial changes can arise as a consequence of pathogenic processes other than a functional iron deficiency, e.g., glucosylsphingosine or galactosylsphingosine toxicity targeted to mitochondrial enzymatic activities [130,170].

Iron deficiency can trigger epigenetic changes that lead to altered expression levels of key proteins (e.g., brain-derived neurotrophic factor, reelin, and cofilin) causing long-term consequences affecting brain development, learning, memory, and plasticity [28,148,149,150,151,154,200,201,202,203]. Plasticity is critical for normal brain development, as well as throughout life, e.g., evolving synaptic connections associated with learning and memory [204]. Iron is necessary for plasticity and proper synaptic function [203,205,206], and plasticity is disrupted in several lysosomal storage disorders [207,208,209]. Therefore, dysfunctional iron homeostasis could contribute to impaired plasticity and learning in lysosomal storage diseases. Since plasticity is also perturbed in Alzheimer’s disease, and this perturbation may even help drive disease progression [210,211,212], it raises the likelihood that a functional iron deficiency is also contributing to impairments of learning and cognition in Alzheimer’s disease in addition to furthering processes leading to neurodegeneration. 

## 5. Concluding Thoughts

There are various ways by which iron, or lack thereof, is thought to contribute to tissue damage. In one category, excess iron has been postulated to be a mediator of neurodegeneration. For example, given that oligodendrocytes and myelin have high levels of iron [72,73], the breakdown of myelin has been proposed to cause an extracellular release of iron, which, in turn, catalyzes reactions leading to oxidative tissue damage [213]. In another potential mechanism, excess iron was found to induce cytosolic aconitase activity, resulting in an increase in the production of glutamate, which could then lead to glutamate excitotoxicity [214]. Ferroptosis has also been proposed to be a pathogenic pathway in Alzheimer’s disease, which has been reviewed extensively elsewhere [215,216]. Here, the opposite of excess iron causing pathology, i.e., a functional deficiency of iron, has been explored as a relevant pathogenic mechanism.

The impact for a functional iron deficiency will likely vary considerably between various lysosomal storage diseases and their subtypes. The range of impacts include: (1) iron transport is unaffected and iron availability is at normal levels for cellular functions, (2) compensatory mechanisms are employed, allowing for iron transport and iron availability to be adequate for cellular functions, (3) compensatory mechanisms are insufficient and a deficiency in the availability of iron leads to the interference of one or more iron-related enzymatic functions, causing a modest contribution to ongoing cellular pathology that can contribute to an overall decline, and (4) a deficiency of available iron causes an impairment of one or more iron-related enzymatic functions, with a distinct contribution to ongoing cellular pathology and overall decline. In aggressive forms of disease, a functional iron deficiency will likely be outweighed or bypassed by other pathogenic mechanisms in these devastating conditions unless a functional deficiency of iron is the main driver of pathology, e.g., the fourth category listed above. In other forms of disease, if they fall into one of the first two categories, then the impact would be absent. If the condition falls in the third category, then a functional iron deficiency could have a compounding effect, culminating in an appreciable impact. For instance, even in the absence of a major biochemical disruption, if the contribution of a functional iron deficiency is small and sustained, or builds over time, neurons may be vulnerable to the added stress of insufficient available iron for use, since they are normally quite active and can be exposed to additional stressors, e.g., at the synapse [217,218]. 

Within neurons, mitochondrial function can be impaired during an iron-deficient (or functional iron-deficient) state, e.g., decreased mitochondrial complex activities [27,36,131]. Alongside limiting the production of ATP and other relevant molecules, dysfunctional mitochondria due to iron deficiency can elevate oxidative damage [6]. Other than impairing mitochondria, the effects of a functional iron deficiency in neurons can include altering dendritic growth, diminishing synaptic function (affecting cognition, learning, and memory), and facilitating neurodegeneration [1,27,28]. In addition to neurons, a functional iron deficiency could affect oligodendrocytes and the activity of myelination, since oligodendrocytes normally have high levels of iron, and a sufficient level of iron is necessary for normal myelination (discussed previously). 

Although the etiologies and the course of disease are different for lysosomal storage diseases and Alzheimer’s disease, there are overlapping downstream pathogenic mechanisms (discussed earlier). The influence of these mechanisms to the disease course is of considerable interest, as they may highlight new therapeutic targets [178,219]. Previously, shared features between these diseases were noted for disrupted autophagy and reduced lysosomal function, e.g., causing the accumulation of gangliosides and impaired digestion of amyloid precursor protein metabolites [178,219]. Here, a functional iron deficiency has been put forward as a potentially common pathogenic mechanism.
